# Antagonistic effects of selenium on lead-induced autophagy by influencing mitochondrial dynamics in the spleen of chickens

**DOI:** 10.18632/oncotarget.16736

**Published:** 2017-03-31

**Authors:** Yujing Han, Chunqiu Li, Mingjun Su, Zhihui Wang, Ning Jiang, Dongbo Sun

**Affiliations:** ^1^ College of Animal Science and Veterinary Medicine, Heilongjiang Bayi Agricultural University, Sartu District, Daqing 163319, P.R. China

**Keywords:** lead, selenium, autophagy, chickens spleen, mitochondrial dynamics

## Abstract

Lead (Pb) may damage the immune function in human and animal. Selenium (Se) has antagonistic effects on Pb. In our study, brown layer chickens were randomly allocated to control group, Se group (1 mg/kg Se), Se+Pb group (1 mg/kg Se and 350 mg/kg Pb), and Pb group (350 mg/kg Pb). The chickens were sacrificed on the 90^th^ day; spleen tissues were subjected to observation of ultrastructure and detection of spleen-related indexes. The results revealed that in the Pb group, expression levels of the cytokines IL-1 and TNF-α significantly increased, and expression levels of IL-2 and INF-γ significantly decreased; activities of antioxidant enzyme GPX, SOD and CAT significantly decreased, and expression level of malondialdehyde (MDA) significantly increased; expression levels of mitochondrial fission-related genes (Mff and Drp1) significantly increased, and expression levels of mitochondrial fusion-related genes (Opa1, Mfn1 and Mfn2) significantly decreased; expression of autophagy-related genes (Beclin 1, Dynein, Atg 5, LC3-I and LC-II) was upregulated, while expression of mammalian target of rapamycin (mTOR) was downregulated. The results of transmission electron microscopy indicated that Pb induced mitochondrial fragmentation, and triggered autophagy in the spleen of chickens. The Se and Pb co-treatment remarkably alleviated these injuries induced by Pb in the spleen of chickens. In conclusion, Pb can induce the oxidative stress to influence the mitochondrial dynamics balance and lead to autophagy, which triggers the immune dysfunction in the spleen of chickens; the Se exhibits the antagonistic effects on lead-induced autophagy by influencing mitochondrial dynamics in the spleen of chickens.

## INTRODUCTION

Lead (Pb) is a well-known highly toxic element for organisms and has the potential to threaten ecosystem. At present, Pb pollution remaining in the soil, air and river is growing into a serious problem for the health of animals and humans [1–3]. Excessive exposure to Pb may induce several pathological changes in humans and animals, involving in encephalopathy, convulsion and even death [4–6]. The oxidative stress and inflammatory are associated with Pb toxicity [7]. Pb can decrease the activities of antioxidant enzymes and cause the altered expression of the cytokines [8–10]. Autophagy is one of the crucial cellular mechanisms for organism, in which mTOR negatively regulates the induction of autophagy [11]. Corsetti et al. (2016) reported that spleen is one of the target organs of Pb toxicity; excessive exposure to Pb may induce autophagy in the spleen [12]; in fishes, spleen exhibits a high content of Pb and histopathological alterations when treated by Pb [13]. Additionally, the evidence convinced that mitochondrial fission could promote mitochondrial autophagy in mouse embryonic fibroblasts, human fibroblasts, and cardiac cells [14–16]; in adult cardiomyocytes, Drp1, a GTPase, regulates mitochondrial fission, and mediates autophagy [17]; the overexpression of the OPA1 decreases mitochondrial autophagy in mice [14]. Accumulating reports indicate that autophagy is one of the most important targets for investigation of mechanisms of Pb toxicity for organism.

Selenium (Se) is an essential trace mineral, involving in cellular antioxidant defense, and anti-inflammatory mechanisms [18–20]. In the chickens, Se obviously attenuates Cd-induced apoptosis in the spleen, thymus, and bursa of Fabricius [21]. In mice, the antagonistic effects of Se on mercury (Hg) have been reported [22]. It has been revealed that dietary Se has antagonistic effects on Pb toxicity [23, 24]. Furthermore, Se has the potential effects on autophagy in organisms [25]; Se deficiency leads to the increased expression of autophagy-related genes in the immune organs of the chickens [26]; Se pretreatment upregulates expression levels of mitochondrial biogenesis regulators, and reduces autophagy in murine hippocampal neuronal HT22 cells [27]. These data demonstrate that Se has the potential antagonistic effects on the toxicity of heavy metals in immune organ.

Although the antagonistic effects of Se on Pb were investigated in humans and rats, there are few studies in the spleen of chickens. In the current study, the interaction model between Pb and Se was established; the ultrastructural changes, antioxidant function, levels of cytokines, autophagy, and expressions of mitochondrial dynamics-related genes were investigated in the Se/Pb-affected chicken spleens, respectively. Our aim was to clarify the alteration of mitochondrial dynamics in Pb toxicity, and explore the potential mechanisms of the antagonistic effects of Se on Pb.

## RESULTS

### Transmission electron microscopy

**Figure 1:**
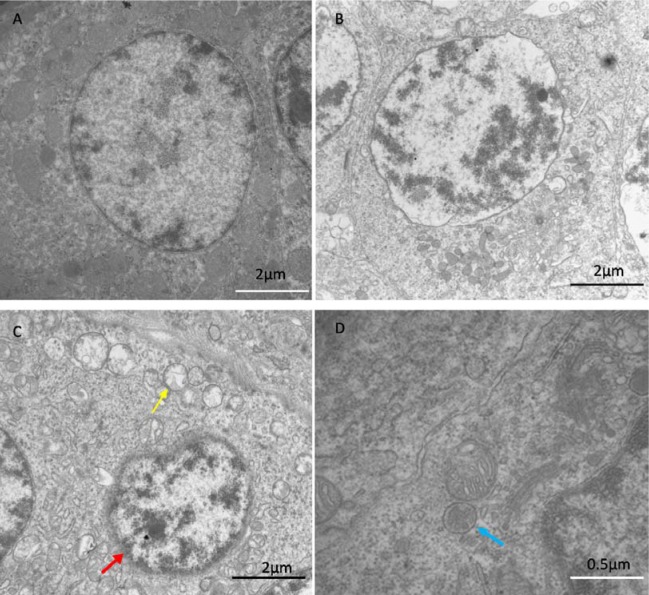
The results of transmission electron microscopy The results showed ultrastructure of spleen in control group **(A)**, Se+Pb group **(B)** and Pb group (**C** and **D**). The Pb group showed mitochondrial vacuole (yellow arrows), chromatic agglutination (red arrow) and the formation of the autophagosome (blue arrows). The pathological injuries in Se+Pb group were almost close to normal level.

In the control group, spleens exhibited the normal ultrastructure with the integrated mitochondria and clear nuclear membrane (Figure 1A). In the Se+Pb group, the pathological injuries were close to the normal levels (Figure 1B). In the Pb group, spleens showed mitochondrial vacuole (yellow arrows), chromatic agglutination (red arrow), and the formation of the autophagosome (blue arrows), suggesting that there were ultrastructural damages in spleen cells (Figure 1C, D).

### Effects of Se, Pb, Se+Pb treatment on the antioxidant markers in the spleen of chickens

**Figure 2:**
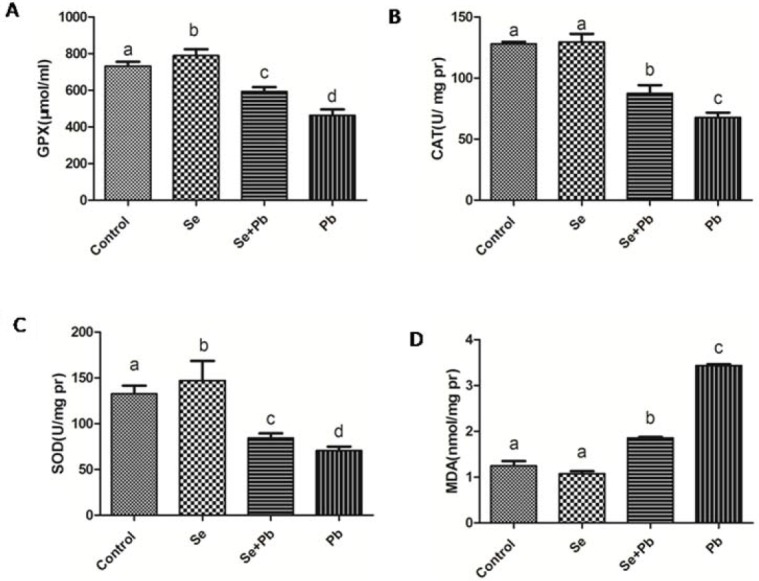
Effects of Se, Pb and Se+Pb treatment on the activities of GPX (A), CAT (B), SOD (C) and MDA (D) level in the spleen of chickens Each value represents the mean±SD (n=6/group). The bars sharing different small letters represent statistically significantly differences between the groups (P < 0.05); the bars with a common letter are not significantly different (P > 0.05).

The results indicated that in the Pb group, the activities of the antioxidant enzymes GPX, CAT and SOD exhibited a significant decrease (P < 0.05), while the expression level of MDA showed a significant increase when compared with other groups (P < 0.05); in the Se group, the activities of the GPX and SOD significantly increased (P < 0.05), but the expression levels of the CAT and MDA had no significant changes when compared with the control group (P > 0.05); in the Se+Pb group, the activities of GPX, CAT and SOD significantly increased and the expression level of MDA significantly decreased when compared with the Pb group (P < 0.05) (Figure 2).

### Effects of Se, Pb and Se+Pb treatment on the expressions of cytokines in the spleen of chickens

**Figure 3:**
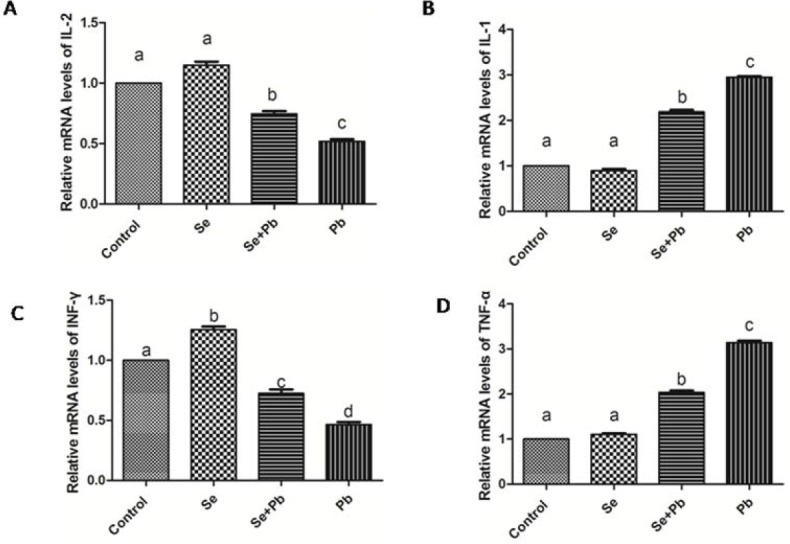
Effects of Se, Pb and Se+Pb treatment on the activities of IL-2 (A), IL-1 (B), INF-γ (C) and TNF-α (D) in the spleen of chickens Each value represents the mean±SD (n=6/group). The bars sharing different small letters represent statistically significantly differences between the groups (P < 0.05); the bars with a common letter are not significantly different (P > 0.05).

In the Se group, the expression levels of the cytokines had no significant changes when compared with the control group (P > 0.05). The decreased expression of IL-2 and INF-γ and the increased expression of IL-1 and TNF-α were found in the Pb group when compared with the control group (P < 0.05). The co-treatment of Se remarkably alleviated this situations when compared with the Pb group (P < 0.05), but did not bring them back to the control level (P > 0.05) (Figure 3).

### Effects of Se, Pb, and Se+Pb treatment on autophagy and expression of the apoptosis-related genes in the spleen of chickens

**Figure 4:**
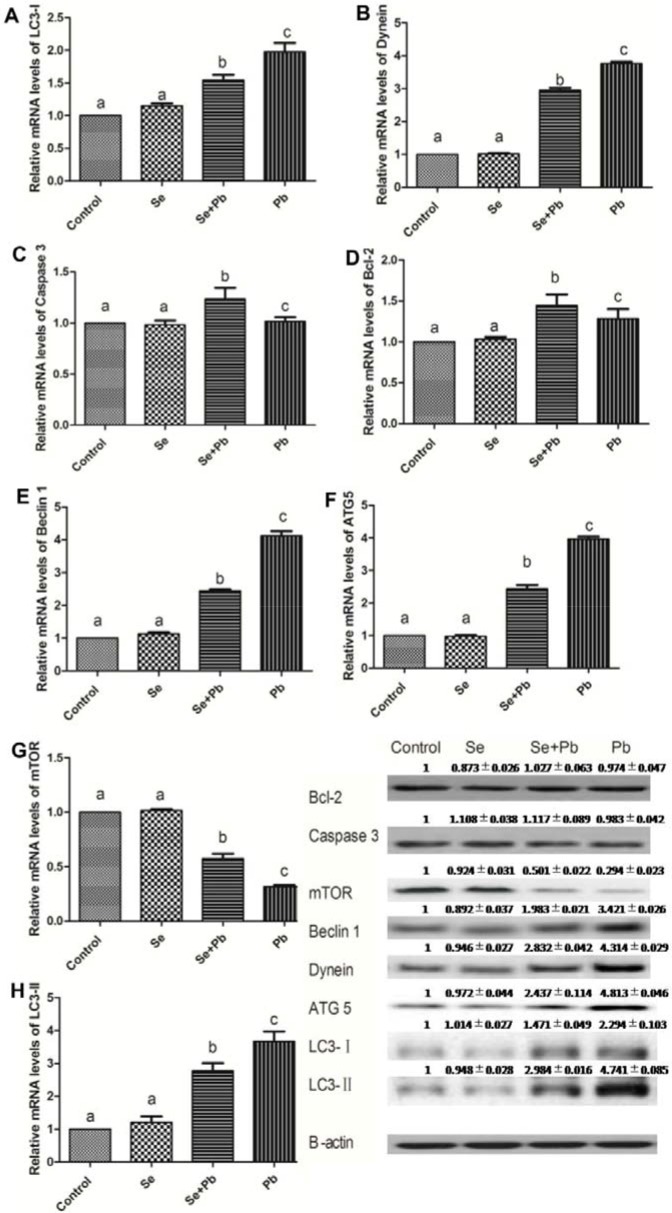
Effects of Se, Pb and Se+Pb treatment on the autophagy and apoptosis-related genes expressions in the spleen of chickens Each value represents the mean±SD (n=6/group). The bars sharing different small letters represent statistically significantly differences between the groups (P < 0.05); the bars with a common letter are not significantly different (P > 0.05).

The results showed that in the Se group, the expression levels of the autophagy/apoptosis-related genes did not exhibit significant difference when compared with the control group (P > 0.05). The Pb treatment up-regulated the expression levels of Bcl-2, Caspase 3, Atg5, Beclin-1, Dynein, LC3-I and LC3-II and down-regulated the expression levels of mTOR when compared with the control group (P < 0.05). The Se+Pb group remarkably alleviated these changes when compared with the Pb group (P < 0.05) (Figure 4).

### Effects of Se, Pb and Se+Pb treatment on expressions of the mitochondrial dynamics-related genes in the spleen of chickens

**Figure 5:**
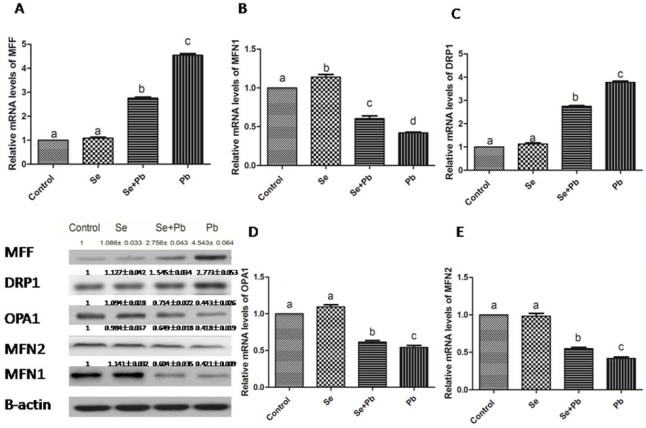
Effects of Se, Pb and Se+Pb treatment on the expressions of MFF (A), MFN1 (B), DRP1 (C), OPA1 (D) and MFN2 (E) in the spleen of chickens Each value represents the mean±SD (n=6/group). The bars sharing different small letters represent statistically significantly differences between the groups P < 0.05); the bars with a common letter are not significantly different (P > 0.05).

In the Se group, the expression levels of the mitochondrial dynamics-related genes did not show significant difference when compared with the control group (P > 0.05). Pb treatment significantly increased the expression levels of MFF and DRP1, and down-regulated the expression levels of MFN1, MFN2 and OPA1 when compared with the control group (P < 0.05). The Se+Pb group remarkably alleviated these situations when compared with Pb group (P < 0.05) (Figure 5).

## DISCUSSION

Pb has been reported to have the ability to damage both macrophages and T cell [28, 29], and may cause immunosuppression or hyperreactivity [30]. The alterated expression of the cytokines is considered to be an indicator of the immunity condition. TNF-α is a cytokine produced by macrophages; IL-1 plays a central role in the regulation of the immune and inflammatory responses; IL-2 has essential roles in key functions of the immune system, primarily via its direct effects on T cells; IFN-γ is closely associated with a number of autoinflammatory and autoimmune diseases; in the plasma of rats, expression levels of IL-1β and TNF-α remarkably increase when treated by Pb [31]; in the bursa of fabricius of the chickens, Pb treatment significantly decreased the expression levels of IL-2 and IFN-γ [32]. Evidences suggested that spleen is one of the target organs of the Pb toxicity, and excessive Pb exposure may cause histological changes of the spleen [33]; Pb administration can induce spleen hypertrophy in mice [12]. In the current study, the expression levels of the cytokines-related genes were investigated in the spleen of chickens treated by Pb. The results indicated that the expression levels of IL-1 and TNF-α significantly increased and the expression levels of IL-2 and IFN-γ significantly decreased. The resulting data in our study indicated effects of the Pb treatment on the immune function of the spleen of chickens, which is in line with the previous studies.

The oxidative stress is one of the representative indexes of the Pb-affected organism. In rats, the activities of GPX and SOD, as oxidative stress markers, significantly decreased in the Pb treatment group [34]. In human, the decreased activities of GPX and SOD were observed when treated by Pb[35]. In the current study, the activities of antioxidative enzymes and the content of MDA were detected, respectively. The results indicated that the Pb treatment leaded to the oxidative stress in the spleen of chickens according to the alterated expression of GPX, SOD, CAT and MDA. The resulting data in our study are consistent with the previous studies. It has been reported that the oxidative stress may trigger apoptosis [36]. In the proximal tubular cells of the Pb-affected rat, the oxidative stress leads to cell apoptosis by the alteration of the mitochondrial permeability transition (MPT) [37]. In our study, the expression levels of the apoptosis-related genes, Caspase 3 and Bcl-2, were detected, respectively. However, the results demonstrated that the treatment of Pb did not exhibit a significant effects on apoptosis. This result suggests that Pb can not enhance the apoptosis in the spleen of chickens. Notably, other reports indicated that oxidative stress was closely associated with autophagy [38, 39]. In H9c2 rat cardiomyoblasts, oxidative stress may suppress mTOR activity and promote autophagy [40]. In addition, previous study documented that Pb toxicity induced autophagy via the mTORC1 pathway in cardio fibroblasts [41, 42]. In our study, the Pb treatment promoted the expressions of Beclin 1, Dynein, ATG 5, LC3-I, and LC3-II and inhibited the expression of the mTOR. Additionally, we also observed the ultrastructure of the spleen of chickens by using transmission electron microscopy. The results showed that there were typical features of autophagy in the Pb group. These data demonstrated that Pb exerted its toxicity in the spleen of chickens by inducing autophagy. Furthermore, the relationship between the autophagy and the mitochondrial fission/fusion has been reported. The number of autophagosomes shows a decrease when the function of Drp1, a GTPase that regulates mitochondrial fission, is blocked [43]. The repression of Mfn2 expression has been revealed to be one determinant factors for the inhibition of autophagy [44]. In our study, the expression levels of mitochondrial fission-related genes significantly increased, while the expression levels of mitochondrial fusion-related genes significantly decreased in the Pb group, which is in agreement with previous studies.

Se, as an essential micronutrient, plays an crucial role in antioxidation and immunity [45–48]. It has been reported that Se has the ability to protect organism against toxicity of heavy metals, including Pb. In Cyprinus carpio, Se exhibits the antagonistic effects on Pb toxicity via antioxidant system [49]. In fish, the altered activities of antioxidant enzymes induced by Pb can be normalized by the Se supplementation [50]. In the liver of chickens, Se shows remission effects on the Pb-induced overexpressions of cytokines[51]. In our study, Se co-treatment decreased the expressions of TNF-α, IL-1 and MDA, increased the activities of SOD, CAT, GPX, and increased the expressions of IL-2 and INF-γ. These data demonstrate that Se can antagonize the toxic effects of Pb by reducing oxidative stress and restoring immune function, which is in keeping with previous study. It has been reported that Se may restore the balance between mitochondrial fission and fusion, and prevent the activation of autophagy [52]. In our study, Se co-treatment increased the expression levels of mitochondrial fusion-related genes, decreased the expression levels of mitochondrial fission and autophagy-related genes. Additionally, it had been confirmed that Se reduced ultrastructural damage induced by Pb treatment in the spleen of chickens. These data elucidate that Se co-treatment alleviates the imbalance of the mitochondrial dynamics and autophagy induced by Pb in the chiken spleen, which is compatile with previous study.

In summary, we conclude that Pb can influence the mitochondrial dynamics balance by inducing the oxidative stress, which leads to autophagy, and finally trigger the immune dysfunction in the spleen of chickens; the Se co-treatment can significantly relieve these injuries induced by Pb.

## MATERIALS AND METHODS

### Animal care and experimental design

The present experiments were in accordance with the Institutional Animal Care and Use Committee of the Heilongjiang Bayi Agricultural University. The experimental animals were purchased from Laboratory Animal Center of Harbin Veterinary Research Institute of the Chinese Academy of Agricultural Sciences. One-day-old brown layer chickens were randomly allocated to control group (basic diet), Se group (diet contained 1 mg/kg Se), Se+Pb group (diet containing 1 mg/kg Se and 350 mg/kg Pb) and Pb group (diet containing 350 mg/kg Pb). All experimental chickens were provided ad libitum consumption of water and food. The chickens were euthanized on the 90^th^ day and the spleens were quickly removed, rinsed with ice-cold sterile deionized water, frozen immediately in liquid nitrogen and stored at−80°C.

### Transmission electron microscopy

The spleen tissues (size: 1.0 mm × 1.0 mm × 10 mm) were fixed in 2.5% glutaraldehyde phosphate-buffered saline (v/v, pH 7.2), and postfixed in 1% osmium tetroxide (v/v). The spleens were stained with 4.8% uranyl acetate. The samples were washed in propylene oxide and impregnated with epoxy resins. The semi-fine sections were contrasted with uranyl acetate and lead citrate for study via microscopy. The microphotographs were taken with a transmission electron microscope (TEM).

### Detection of the antioxidative enzymes and MDA

The activities of SOD, CAT and GPX and MDA level in the spleen of chickens were detected by kits (Nanjing Jiancheng Bioengineering Institute, PR China) according to the manufacturer's instructions. All samples were detected in duplicate in a single assay.

### Real-time PCR

Total RNAs of the spleen in each group were extracted by TRIzol reagent (Invitrogen, China). The content and purity of the RNA were measured spectrophotometrically at 260/ 280 nm. First-strand complementary DNA was synthesized according to the manufacturer's protocol (Invitrogen, China), and then was stored at −80°C for PCR.

Specific primers of the target genes were designed by uing Primer Premier Software 5.0 (PREMIER Biosoft International, USA) (Table 1). The β-actin was used as an internal reference. Quantitative real-time PCR reactions were run in a 20μL reaction mixture using an ABI 7500 Detection System (Applied Biosystems, USA). Each RT reaction was comprised of 10 μl of 2× SYBR Green II PCR Master Mix (TaKaRa, China), 0.4 μl of each primer (10 μM), 0.4 μl of 50× ROX reference Dye II, 2 μl of cDNA, and 6.8μl of PCR-grade water. The PCR procedure consisted of 95°C for 30 s followed by 40 cycles of 95°C for 5 s, 60°C for 34 s. The mRNA relative abundance was determined by using the method of Pfaffl [53].

**Table 1:** Gene-special primers for qPCR

Gene	Forward Primer	Reverse primer
β-actin	5-CCGCTCTATGAAGGCTACGC-3	5-CTCTCGGCTGTGGTGGTGAA-3
IL-1	5-CTCCTCCAGCCAGAAAGTGA-3	5-GAGCTTGTAGCCCTTGATGC-3
IL-2	5-TGCAGTGTTACCTGGGAGAA-3	5-CGGTGTGATTTAGACCCGTAA-3
IFN-γ	5-AGCCGCACATCAAACACATA-3	5-CGCTGGATTCTCAAGTCGTT-3
TNF-α	5-AGATGGGAAGGGAATGAACC-3	5-ACTGGGCGGTCATAGAACAG-3
Mff	5-TGGGAAGGCTGAAGAGAGAA-3	5-GGTGTTCCCTCAAGTGTGGT-3
Drp1	5-GGCAGTCACAGCAGCTAACA-3	5-GCATCCATGAGATCCAGCTT-3
Opa1	5-GCTACGGACCAGGGTTATGA-3	5-GCTCAAGCATCCGTTGGTAT-3
Mfn2	5-TACCAGGCAGATTTCCATCC-3	5-GTGATTGCATTGGAACAACG-3
Mfn1	5-TGAGCATGTAGCAACGGAAG-3	5-AGCAAGCTGATTGACGGTCT-3
Bcl-2	5-ATCGTCGCCTTCTTCGAGTT-3	5-ATCCCATCCTCCGTTGTCCT-3
Caspase3	5-CATCT GCATCC GTGCCTGA-3	5-CTCTCGG CTGTGGTGGTGAA-3
LC3-I	5-GCATCCAAACAAAATCCCAGTC-3	5-AAGCCATCCTCATCCTTCTCCT-3
LC3-II	5-CTTCTTCCTCCTGGTGAACG-3	5-GCACTCCGAAAGTCTCCTGA-3
Dynein	5-TGGGATAATCGCAGCAATAAGA-3	5-AGGGAAGGACATGCAAGTAACAG-3
Beclin-1	5-CAGACACGCTGCTGGACC-3	5-TCTCCTTGTCATCCTCGTTCA-3
Atg 5	5-GATGAAATAACTGAAAGGGAAGC-3	5-TGAAGATCAAAGAGCAAACCAA-3
mTOR	5-GAAGAGCTGATTCGGGTAG-3	5- ACCATTCTTGTGCCTCCATT-3

### Analysis of western blot

Equal equivalent amount of the total protein (40μg/condition) were separated by SDS-polyacrylamide gel electrophoresis under reducing conditions. The separated proteins were transferred to nitrocellulose membranes by a tank transfer for 2 h at 200 mA in Tris–glycine buffer containing 20 % methanol. Membranes were blocked with 5 % skim milk for 24 h and incubated overnight with primary antibody against Bcl-2, Caspase-3, Drp-1, Mff, Mfn1, Mfn2, Opa1, mTOR, Beclin 1, Dynein, Atg5, LC3-I and LC3-II (Santa Cruz Biotechnologies, CA, USA). After the final wash, the blots were incubated with the horse-radish peroxidase (HRP) conjugated secondary antibodies (Santa Cruz, USA). The membrane was incubated with a monoclonal β-actin antibody (1:1000, Santa Cruz, USA), followed by incubation with a HRP conjugated goat anti-mouse IgG (1:1000). The protein bands were visualized by enhanced chemiluminescence detection reagents (Applygen Technologies Inc., Beijing, China). The signal was detected using X-ray films (TransGen Biotech Co., Beijing, China). The optical density of each band was determined by using an Image VCD gel imaging system (Beijing Sage Creation Science And Technology Co. Ltd., Beijing, China), and the relative abundance of the proteins were expressed as the ratios of OD of each of these proteins to that of β-action.

### Statistical analysis

Data was analyzed by SPSS software (Windows version 21.0; SPSS Inc., Chicago, IL). One-way analysis of variance followed by the Tukey honest significant difference test was used to analyze the descriptive statistics (mean values, standard deviation). P < 0.05 was considered to be significant.

## Abbreviations

COX-2: cyclooxygenase-2
MPT: mitochondrial permeability transition
GPX: glutathione peroxidase
SOD: superoxide dismutase
CAT: catalase
MDA: malondialdehyde
IL-2: interleukin 2
IL-1: interleukin 1
INF-γ: interferon gamma
TNF-α: tumor necrosis factor alpha
Mff: mitochondrial fission factor
Drp1: dynamin related protein 1
Opa1: mitochondrial dynamin like GTPase
Mfn 1: mitofusin 1
Mfn 2: mitofusin2. Bcl-2, B-cell lymphoma-2
Caspsase 3: cysteinyl aspartate specific proteinase
mTOR: mechanistic target of rapamycin
Atg 5: autophagy-related gene-5
LC3-I: microtubule-associated protein 1 light chain 3 alpha
